# Incense smoke-induced oxidative stress disrupts tight junctions and bronchial epithelial barrier integrity and induces airway hyperresponsiveness in mouse lungs

**DOI:** 10.1038/s41598-021-86745-7

**Published:** 2021-03-31

**Authors:** Norio Yamamoto, Keiko Kan-o, Miyoko Tatsuta, Yumiko Ishii, Tomohiro Ogawa, Seiji Shinozaki, Satoru Fukuyama, Yoichi Nakanishi, Koichiro Matsumoto

**Affiliations:** 1 Research Institute for Diseases of the Chest, Graduate School of Medical Sciences, Kyushu University, 3-1-1 Maidashi, Higashi-ku, Fukuoka, 812-8582 Japan; 2 Department of Endoscopic Diagnostics and Therapeutics, Kyushu University Hospital, Fukuoka, Japan; 3 Department of Respiratory Medicine, National Hospital Organization Omuta National Hospital, Fukuoka, Japan

**Keywords:** Respiratory tract diseases, Experimental models of disease

## Abstract

Recent clinical studies have suggested that inhalation of incense smoke (IS) may result in impaired lung function and asthma. However, there is little experimental evidence to link IS with airway hyperresponsiveness (AHR) and bronchial epithelial barrier function. Using mouse and cell culture models, we evaluated the effects of IS exposure on AHR, expression of multiple epithelial tight junction (TJ)- and adherens junction-associated mRNAs and proteins in the lungs, and the barrier function of bronchial epithelial cells assessed by transepithelial electronic resistance (TEER). Exposure of BALB/c mice to IS increased AHR and inflammatory macrophage recruitment to BALF; reduced claudin-1, -2, -3, -7, -10b, -12, -15, and -18, occludin, zonula occludens-1 [ZO-1], and E-cadherin mRNA expression; and caused discontinuity of claudin-2 and ZO-1 protein immunostaining in lung tissue. IS extract dose-dependently decreased TEER and increased reactive oxygen species production in bronchial epithelial cell cultures. Treatment with *N*-acetyl-l-cysteine, but not glucocorticosteroids or long-acting β_2_-agonists, prevented the detrimental effects of IS. IS exposure can be problematic for respiratory health, as evidenced by AHR, increased recruitment of inflammatory macrophages and disruption of TJ proteins in the lung, and damage to epithelial barrier function. However, antioxidants may be useful for the treatment of IS-induced airway dysfunction.

## Introduction

Burning of incense in temples and homes is a common religious and cultural practice in many Asian and Middle Eastern countries. Incense smoke (IS) contains particulate matter of varying sizes; gases such as carbon monoxide, nitrogen dioxide and sulfur dioxide; and volatile organic compounds such as benzene, aldehydes and polycyclic aromatic hydrocarbons^1^. Compared with cigarette smoking, incense burning has been reported to generate larger quantities of particulate matter of ≤ 2.5 μm diameter and particles that remain in the air for hours^2,3^. Increasing evidence suggests that ambient air pollution from IS can cause health problems, especially airway dysfunction. Recent studies have reported that indoor exposure to IS increases the risk of wheezing and asthma and is associated with impaired lung function in adolescents^4–7^. Lin et al*.* showed that incense smoke exposure during pregnancy is a risk factor for elevated serum immunoglobulin E (IgE) in human umbilical cord blood^8^. Despite a number of clinical studies supporting increased risk of asthma, however, there is little experimental evidence that inhalation of IS causes airway dysfunction.

Bronchial epithelial cells form a barrier against a wide range of inhaled substances and are at the front line of mucosal innate immunity. The epithelial barrier function is maintained by apical junctional protein complexes, composed of apical tight junctions (TJs) and underlying adherens junctions (AJs), that form between neighbouring cells^9^. TJ-associated proteins, which include members of the occludin, claudin, and junctional adhesion molecule families, and AJ-associated proteins such as E-cadherin are the major constituents of junctional complexes in the bronchial epithelium^10^. Members of the zonula occludens (ZO) protein family act as scaffolds that link the intracellular domains of TJ and AJ-associated proteins with the cytoskeleton. There is increasing evidence that reduced junctional protein expression and airway barrier function are observed in patients with asthma that may contribute to the development of asthma by enabling greater penetration of inhaled allergen and noxious particles^11,12^. We recently reported that exposure of human bronchial epithelial cells to cigarette smoke in vitro disrupted epithelial barrier function and simultaneously downregulated the expression of multiple TJ and AJ-associated proteins^13^. However, the effects of inhalation of harmful substances on multiple junctional protein expression in animal models is unknown.

In this study, we evaluated the effect of a single exposure of mice to IS on airway hyperresponsiveness (AHR), inflammation and multiple TJ and AJ-associated protein expression in the lung, and we additionally analysed the effect of exposure to IS extract (ISE) on epithelial barrier function in human bronchial epithelial cells in air–liquid interface (ALI) cultures. The benefits of inhaled glucocorticosteroids (GCSs) and long-acting β_2_-agonists (LABAs) in asthma are widely recognized^14^. Therefore, we also investigated whether treatment with GCSs, LABAs, or the antioxidant *N*-acetyl-l-cysteine (NAC) could protect against IS-induced airway dysfunction in vitro and in vivo.

## Methods

### Mice

Six-week-old female BALB/c mice were purchased from Japan SLC (Shizuoka, Japan) and housed under specific pathogen-free conditions. The study protocol was approved by The Kyushu University Animal Care and Use Committee (A19-200-0). All experiments were performed in accordance with our institutional guidelines and carried out in compliance with the ARRIVE guidelines.

### Mouse experiments

The most commonly used incense sticks in Japan were used for the study (0.4 g/stick, Nippon Kodo brand). Mice were randomly assigned to two or three groups depending on the experimental protocol: (i) unexposed, (ii) exposed to a high dose of IS (IS^high^), or (iii) exposed to a low dose of IS (IS^low^). The mouse groups were housed separately to avoid cross-exposure to IS. Mice were placed in 44-L chambers and exposed to IS generated by burning of 3.2 g (IS^high^) or 1.6 g (IS^low^) of incense sticks for 60 min. Incense sticks were burnt in a separate 8-L chamber connected by a tube to the 44-L exposure chamber, and IS was drawn into the exposure chamber with fresh air at 4 L/min. Mice from the unexposed group were maintained for the same period in fresh air. The ratio of the exposure chamber volume to the mean body weight of mice was designed to recapitulate the ratio of a standard living room volume to the standard body weight of humans.

In some experiments, mice received intraperitoneal injections of NAC (320 mg/kg, Sigma-Aldrich, St. Louis, MO) at 6 h before and 6 h after the 1-h exposure to IS. NAC was dissolved in phosphate buffer saline (PBS) and neutralised to pH 7.4 with NaOH.

### ISE preparation

ISE for in vitro experiments was prepared by bubbling IS from 1.6 g of incense sticks (Nippon Kodo brand) through 20 mL of culture medium. After adjustment of the pH to 7.4, the ISE was sterile-filtered (0.22-μm pore size, 33-mm diameter Millex GV; Merck Millipore, Billerica, MA). This solution was considered to be 100% ISE and was diluted in medium containing 10% foetal bovine serum (FBS) before use. ISE preparations were standardized based on absorbance at 320 nm and were freshly prepared for each experiment.

### Measurement of AHR

Airway responsiveness was measured according to our previously described protocol^15^. Briefly, mice were anesthetized with a mixture of ketamine and sodium pentobarbital intraperitoneally, and then their tracheas were cannulated via tracheostomy. Mice were ventilated mechanically (tidal volume, 0.3 mL; frequency, 120 breaths/min) after a paralytic agent was administered. The airway opening pressure was measured with a differential pressure transducer and recorded continuously. Stepwise increase in acetylcholine dose (1.25–20 mg/mL) were given with an ultrasonic nebulizer (NE-U07; OMRON Co., Kyoto, Japan) for one minute. The data were expressed as the provocative concentration 200 (PC_200_), the concentration at which airway pressure was 200% of its baseline value. PC_200_ was calculated by log-linear interpolation for individual animals as described previously^15^. Values of PC_200_ were expressed as log (100 × PC_200_).

### Collection of bronchoalveolar lavage fluid (BALF), flow cytometric analysis

After measurement of AHR, mice were euthanised by administration of pentobarbital. For collection of BALF, the lungs were gently lavaged twice with 1 mL of 0.9% saline via a tracheal cannula. BALF was centrifuged at 250 × *g* for 10 min and then total and differential cell counts in BALF were performed as described previously^16^.

For flow cytometry analysis, lungs not subjected to bronchoalveolar lavage were minced and single-cell suspensions were prepared. Macrophages and inflammatory macrophages were identified and enumerated by flow cytometry (Becton Dickinson, Franklin Lakes, NJ), as described in detail in the Supplementary Methods.

### Fluorometric TUNEL assay

Apoptotic cells in lung tissue sections were detected using a DeadEnd Fluorometric TUNEL System (Promega, Madison, WI) according to the manufacturer’s instructions and observed by confocal laser microscopy (LSM700; Zeiss, Jena, Germany). Negative and positive control slides were prepared by omitting the TdT enzyme from the nucleotide mix and by treating tissue sections with DNase I, respectively.

### Quantitative reverse-transcription PCR (qRT-PCR)

Total RNA was extracted from mouse lungs, reverse-transcribed, and subjected to qPCR as described in the Supplementary Methods. Primer sequences are provided in Supplementary Table S1.

### Immunofluorescence staining

Immunofluorescence staining were performed according to modified protocol as described previously^17^. Briefly, freshly isolated lungs were washed in PBS and then lung tissue was embedded using optimal cutting temperature compound and frozen. Four μm tissue sections on microscope slides were fixed with cold methanol for 10 min at − 20 °C and then blocked with PBS containing 1% BSA for 30 min at room temperature. The tissue sections were incubated with each primary antibody prepared in PBS containing 1% BSA at 4 °C overnight, followed by incubation with Alexa Fluor 488-conjugated goat anti-rabbit IgG antibody (diluted 1:500; Abcam, Cambridge, UK), Alexa Fluor 568-conjugated goat anti-rat IgG antibody (diluted 1:500; Abcam) and nuclear staining with 4′,6-diamidino-2-phenylindole (DAPI). Images of the stained tissue sections were obtained with a confocal laser microscope (LSM700; Zeiss). The primary antibodies were as follows: rabbit anti-claudin-1 polyclonal antibody (diluted 1:200; Thermo Fisher Scientific, Waltham, MA); rabbit anti-claudin-2 polyclonal antibody (diluted 1:200; Abcam); rabbit anti-claudin-10 polyclonal antibody (diluted 1:200, Thermo Fisher Scientific); rabbit anti-claudin-15 polyclonal antibody (diluted 1:200; Thermo Fisher Scientific); rabbit anti-occludin polyclonal antibody (diluted 1:200; Thermo Fisher Scientific); rat anti-ZO-1 monoclonal antibody (diluted 1:200; Santa Cruz Biotechnology, Inc., Santa Cruz, CA); and rabbit anti-E-cadherin polyclonal antibody (diluted 1:200; Thermo Fisher Scientific). Immunofluorescence intensity of TJ and AJ-associated proteins and DAPI was quantified using ImageJ and the relative quantity of TJ and AJ-associated proteins was plotted against DAPI and normalized by unexposed control.

### Cell culture and treatment

Calu-3 cells, a sub-bronchial human epithelial cell line (HTB-55; American Type Culture Collection, Manassas, VA) were cultured in Dulbecco’s modified Eagle’s medium/F-12 (Thermo Fisher Scientific) supplemented with 10% FBS and 1% penicillin–streptomycin and maintained at 37 °C in a humidified atmosphere of 5% CO_2_ in air. The methods for ALI cultures are described in the Supplementary Methods. Preliminary experiments demonstrated that transepithelial electronic resistance (TEER) of the cells cultured under ALI conditions reached a plateau at about day 8 post-seeding and then decreased to about half-maximal levels at day 21^13^.

For the present study, cells were cultured for 8 days, and on day 9, 200 μL of control medium, ISE, and/or pharmacological reagents diluted to appropriate final concentration in culture medium supplemented with 10% FBS were added to the apical chamber and the cells were cultured for the indicated times. For experiments with GCSs, LABAs, or NAC, cells were pretreated with 10 nM fluticasone propionate (FP; Tocris, Minneapolis, MN), 10 nM salmeterol (SAL; Tocris), 10 nM budesonide (BUD; Sigma-Aldrich), 10 nM formoterol (FOR; Sigma-Aldrich), or 1 mM NAC (Sigma-Aldrich) by addition to the apical and basal chambers for 2 h before addition of ISE. Drugs were present throughout the remainder of the incubation. The concentration of GCSs used reflects the estimated therapeutic levels achieved in the human lung during inhalation^18^.

### Measurement of TEER

Bronchial epithelial cell layer integrity was evaluated by TEER measurements using a Millicell-ERS 2V-Ohmmeter (Millipore Co., Bedford, MA). Medium was added to the apical chamber 1 h prior to TEER measurement. The electrode was soaked in 70% ethanol and rinsed with culture medium prior to use. TEER was calculated by the following equation^19^: TEER (Ω cm^2^) = (R_sample_ − R_blank_) × effective membrane area (cm^2^).

### Permeability assay

Calu-3 cell permeability was assessed using a fluorescein isothiocyanate (FITC)-dextran (4 kDa, Sigma-Aldrich) assay as described in the Supplementary Methods.

### Viability assay

Cell viability was assessed according to our previously described protocol^13^. After 24 h of ISE exposure, the medium in the apical chamber was removed and Calu-3 cells were washed with PBS, detached with trypsin–EDTA, and stained with 0.4% trypan blue solution. Non-viable cells were counted in a LUNA Automated Cell Counter (Logos Biosystems, Annandale, VA).

### Reactive oxygen species (ROS) assay

Calu-3 cells were grown in monolayer culture to approximately 50% confluency, incubated with 1 mM NAC or vehicle for 2 h and then incubated with or without 50% ISE for 1 h. Positive control cells were exposed to 200 μM of the ROS inducer pyocyanin (10 mM stock in anhydrous dimethylformamide; Enzo Life Sciences, Farmingdale, NY). Total ROS and superoxide production were evaluated using a ROS-ID Total ROS/Superoxide detection kit (Enzo Life Sciences) according to the manufacturer’s instructions. The cells were then visualised by fluorescence microscopy (BZ-X800; KEYENCE, Osaka, Japan).

### Statistical analysis

Unless otherwise stated, data are expressed as the mean ± standard error (SEM). The Mann–Whitney U-test was used to compare data between two groups and one- or two-way analysis of variance (ANOVA) followed by Tukey's multiple comparisons test were used to compare three or more datasets. Statistical analyses were conducted with Prism 8 software (GraphPad Software, San Diego, CA). Differences were considered statistically significant at *p* < 0.05.

## Results

### IS exposure induces airway hyperresponsiveness and inflammation in mouse lungs

**Figure 1:**
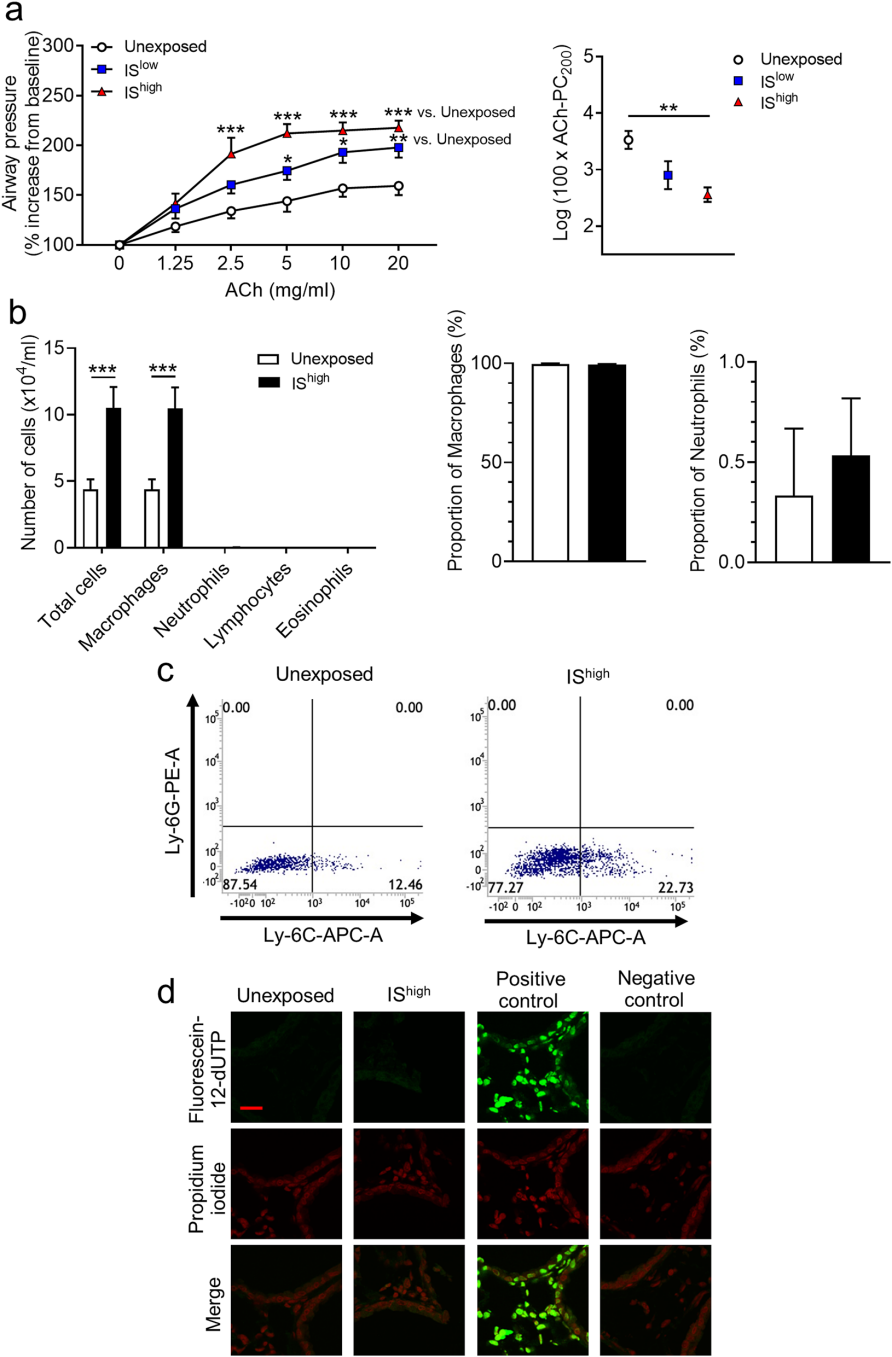
Effects of IS exposure on airway responsiveness, inflammation, and cell apoptosis in mouse lungs. Mice were unexposed or exposed for 1 h to high or low doses of IS and evaluated 24 h later. (**a**) Acetylcholine-induced airway hyperresponsiveness and (**b**) Total and differential cell counts and percentage of cells in BALF. Data are presented as the mean ± SEM (n = 7–14 per group) and were pooled from three independent experiments. *p < 0.05, **p < 0.01, ***p < 0.001 by the Mann–Whitney U-test or one- or two-way ANOVA as appropriate. (**c**) Flow cytometry analysis of macrophage subsets in lung-tissue-derived cells and (**d**) Fluorometric TUNEL assay of apoptotic (green) cells in lung sections. DNA was stained with propidium iodide (red). Scale bar, 20 μm. Results are representative of two independent experiments (n = 3 in each group per experiment). *ACh* acetylcholine, *IS* incense smoke, *PC*_*200*_ provocative concentration causing 200% response.

To determine the effects of a single exposure period to IS on airway function, groups of mice were exposed to fresh air (unexposed) or high or low doses of IS for 1 h, and AHR, recruitment of inflammatory cells to BALF, and apoptosis of lung cells were assessed over the following 24 h. IS exposure increased AHR, as reflected by PC_200_ values, in an acetylcholine dose-dependent manner (Fig. 1a). The number of macrophages, but not neutrophils, lymphocytes or eosinophils, in BALF was also significantly increased in IS-exposed compared with unexposed mice (Fig. 1b). There was no significant difference in the proportion of macrophages and neutrophils in BALF between the two groups (Fig. 1b). Lymphocytes and eosinophils were observed only in BALF from IS-exposed mice (mean percentage of cells: 0.13%, 0.08%, respectively). Flow cytometry analysis of lung-derived cells showed that IS exposure specifically increased the Ly-6G^low^/Ly-6C^high^ population of inflammatory macrophages, but not neutrophils (Fig. 1c and Supplementary Fig. S1). Analysis of apoptosis in lung sections using a fluorometric TUNEL assay revealed no increase in cell apoptosis in the lungs of IS-exposed compared with unexposed mice (Fig. 1d).

### IS exposure decreases expression of TJ- and AJ-associated genes in mouse lungs

**Figure 2:**
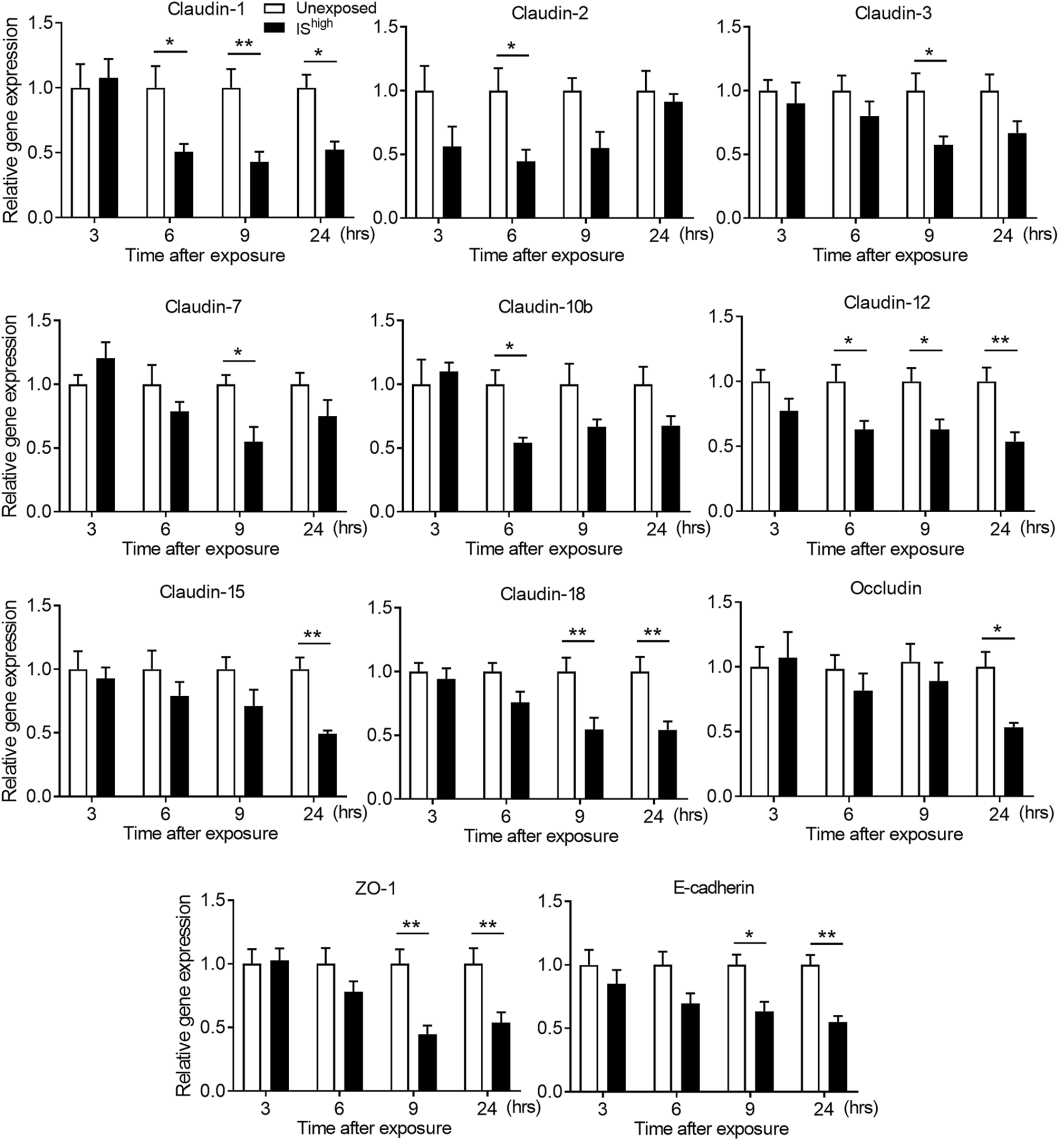
Effects of IS exposure on TJ- and AJ-associated gene expression in mouse lungs. qRT-PCR analyses of the indicated mRNAs in lung tissues was performed at up to 24 h after a 1-h exposure to fresh air (unexposed) or IS^high^. mRNA levels were normalised to GAPDH mRNA levels. Data represent the mean ± SEM (n = 6–9 per group) and were pooled from two independent experiments. *p < 0.05, **p < 0.01, by two-way ANOVA. *IS* incense smoke.

Next, we performed qRT-PCR analysis of lung tissues to determine the effects of IS inhalation on transcription of TJ-associated (claudins, occludin and ZO-1) and AJ-associated (E-cadherin) genes in mouse lungs. IS exposure significantly decreased the mRNA levels of claudin-1, claudin-2, claudin-10b and claudin-12 at 6 h post-exposure; claudin-3, claudin-7, claudin-18, E-cadherin and ZO-1 at 9 h post-exposure; and claudin-15 and occludin at 24 h post-exposure (Fig. 2). Claudin-2, claudin-3 and claudin-7 mRNA levels returned to unexposed control levels by 24 h (Fig. 2).

### Expression of claudin-2 and ZO-1 protein in mouse lungs is disrupted by IS exposure

**Figure 3:**
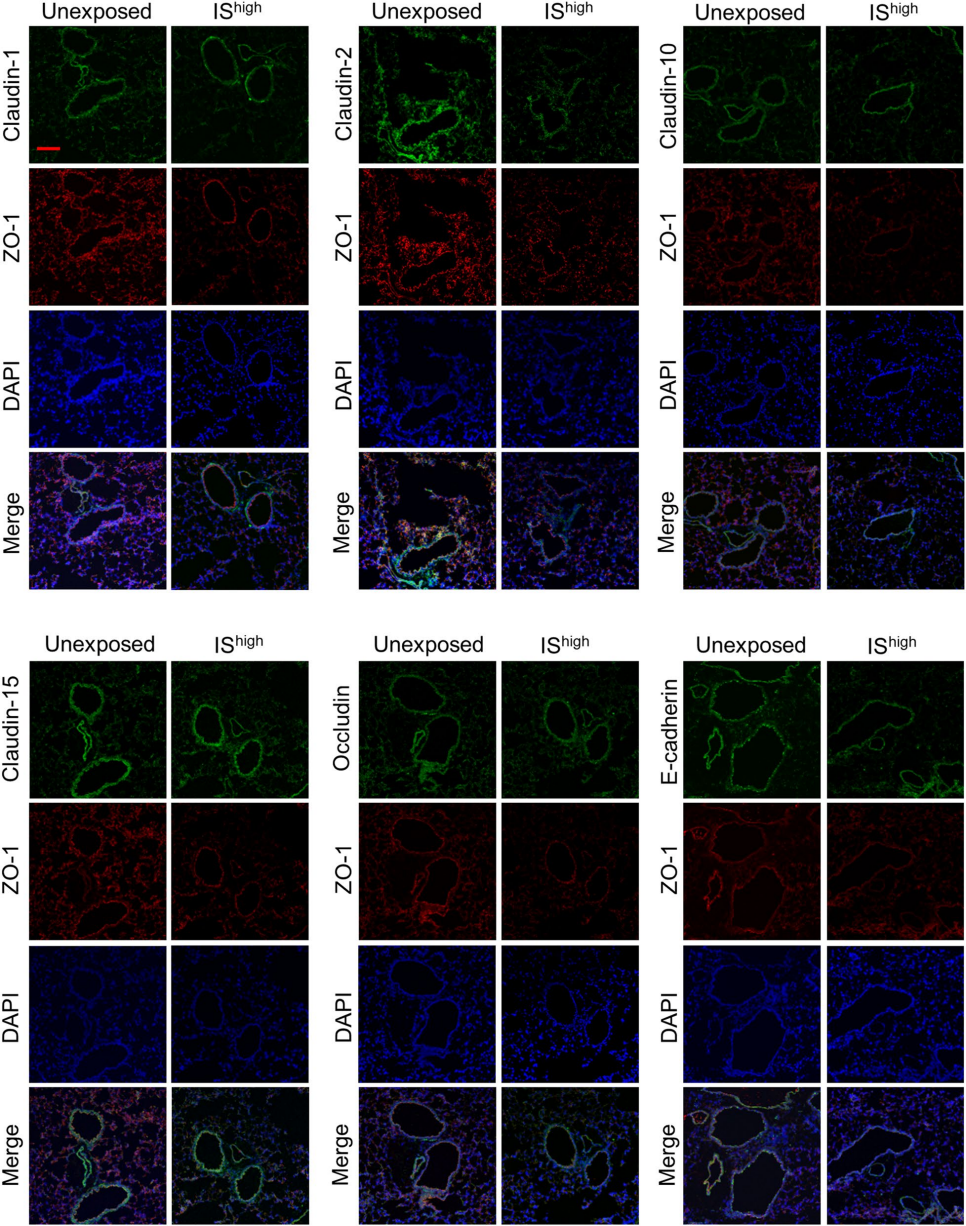
IS exposure-induced changes in TJ and AJ-associated protein expression in mouse lungs. Confocal immunofluorescence microscopy of ZO-1 (red) and additional AJ and TJ-associated proteins (green) in lung sections was performed at 24 h after a 1-h exposure to fresh air (unexposed) or IS^high^. DAPI staining of nuclei is shown in blue. Scale bar, 100 μm. Results are representative of two independent experiments (n = 3 in each group per experiment). *DAPI* 4′,6-diamidino-2-phenylindole, *IS* incense smoke.

To determine whether disruption of TJ- and AJ-associated genes was also observed at the protein level, we performed immunofluorescence staining of lung sections from unexposed and IS^high^-exposed mice. Confocal microscopy revealed predominant staining of claudin-1, claudin-10 and E-cadherin in the bronchial epithelium of unexposed mice, while claudin-2, claudin-15, occludin and ZO-1 proteins were expressed in both bronchial epithelial and alveolar cells (Fig. 3 and Supplementary Fig. S2). Notably, lung sections from IS^high^ mice showed discontinuous or reduced staining of claudin-2 and ZO-1 at 24 h after IS exposure, indicating disruption of TJs (Fig. 3). Immunofluorescence intensity of claudin-2 and ZO-1 was significantly decreased in IS^high^-exposed compared with unexposed mice (Supplementary Fig. S3).

### IS impairs bronchial epithelial barrier integrity and increases epithelial permeability

**Figure 4:**
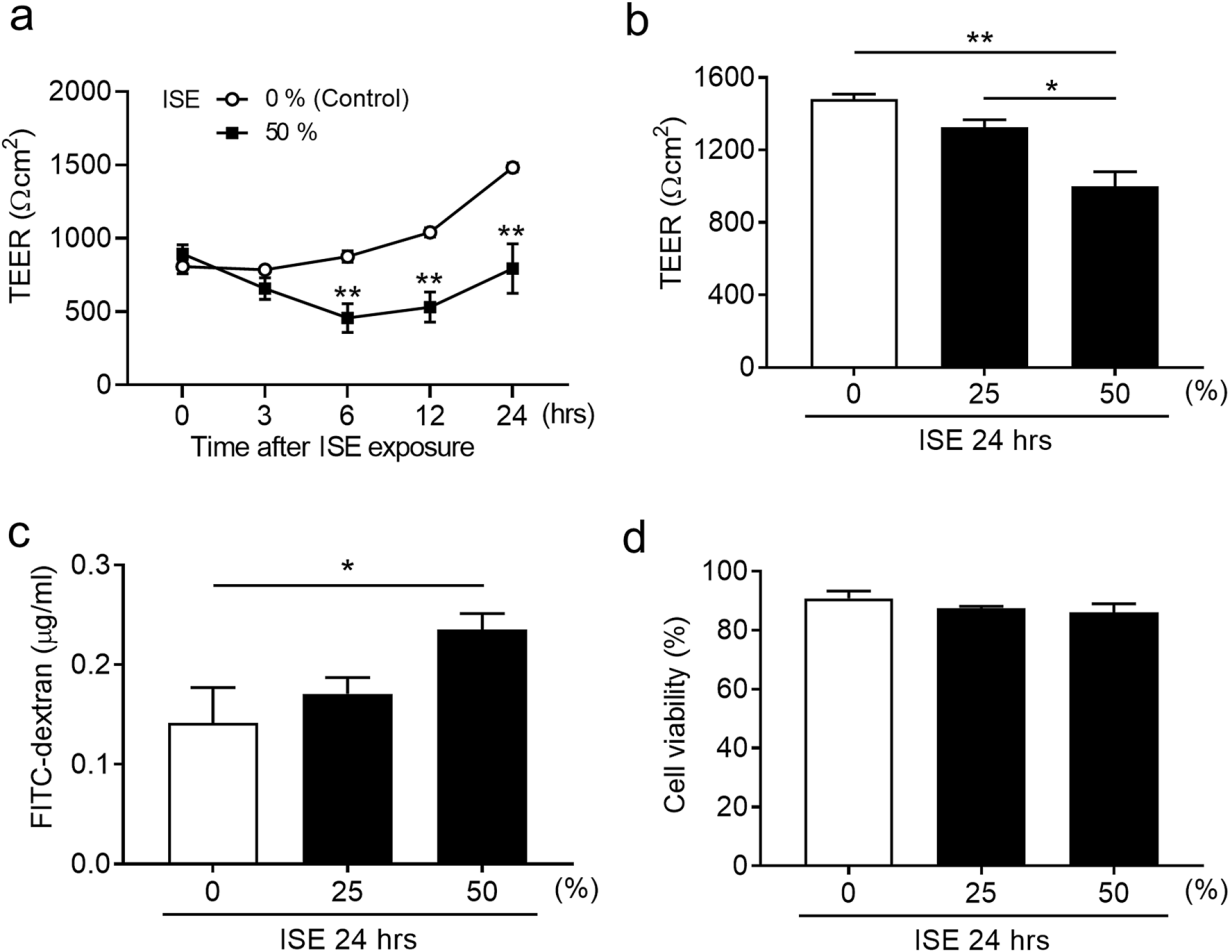
Effects of ISE on airway epithelial barrier function. (**a**) Time course of TEER in Calu-3 cells cultured under ALI conditions for the indicated times after exposure to vehicle (0%) or 50%ISE at the indicated concentrations. (**b**) TEER measured at 24 h after exposure of Calu-3 cells to 0–50% ISE for 24 h. (**c**) FITC-dextran permeability assay of Calu-3 cells after exposure to 0–50% ISE for 24 h. (**d**) Trypan blue viability assay of Calu-3 cells after exposure to 0–50% ISE for 24 h. Data represent the mean ± SEM (n = 6–10 per group) and were pooled from three independent experiments. *p < 0.05, ** *p* < 0.001 by one- or two-way ANOVA as appropriate. *ISE* incense smoke extract, *TEER* transepithelial electrical resistance.

TEER measurements and permeability assays were performed to assess the effects of IS on epithelial barrier function. Calu-3 sub-bronchial epithelial cells were differentiated in ALI cultures for 9 days, and then incubated with 0%, 25%, or 50% ISE concentrations for an additional 24 h. Treatment with 50% ISE caused a reduction in TEER that was maintained for 24 h post-exposure (Fig. 4a). In contrast to the significant reduction in TEER and increases in permeability induced by 50% ISE (Fig. 4b,c), the viability of Calu-3 cells was not influenced at 24 h by exposure to 50% ISE (Fig. 4d). Thus, treatment with 50% ISE was selected for outcome measurements in subsequent experiments.

### ISE-induced impairment of bronchial epithelial barrier integrity is not affected by GCS or LABA treatment

**Figure 5:**
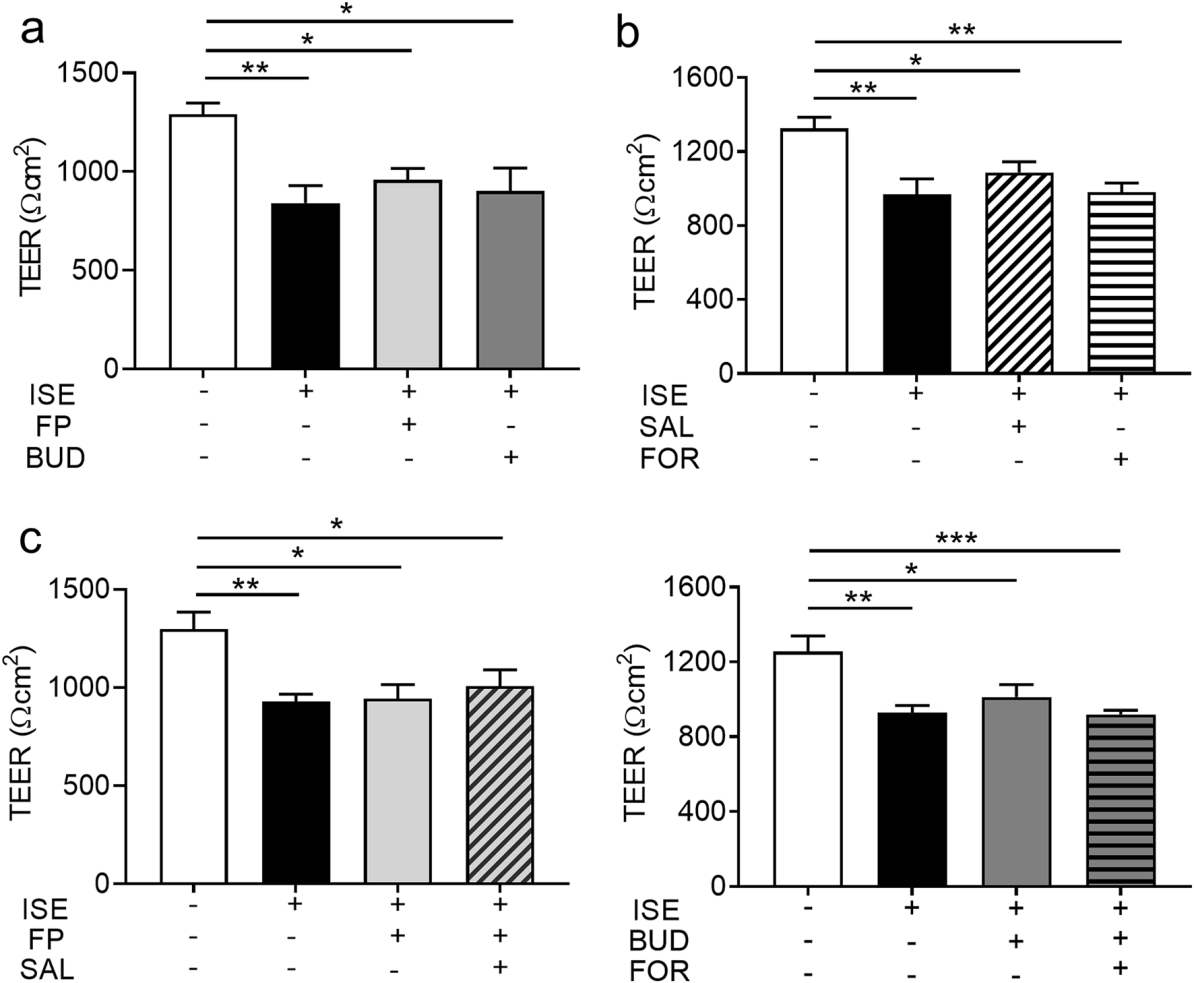
Effects of GCS and/or LABA treatment on ISE-induced reduction in TEER in Calu-3 cells. Cells were cultured under ALI conditions and pretreated with vehicle, 10 nM GCS and/or 10 nM LABA for 2 h prior to addition of vehicle or 50% ISE for 24 h. (**a**) Cells incubated with or without 10 nM of the GSCs FP and BUD. (**b**) Cells incubated with or without 10 nM of the LABAs SAL and FOR. (**c**) Cells incubated with the indicated combinations of 10 nM GCSs and 10 nM LABAs. Data represent the mean ± SEM (n = 5–10 per group) and were pooled from three independent experiments. *p < 0.05, **p < 0.01, ***p < 0.001 by one-way ANOVA. *BUD* budesonide, *FP* fluticasone propionate, *FOR* formoterol, *ISE* incense smoke extract, *SAL* salmeterol, *TEER* transepithelial electrical resistance.

We previously demonstrated that GCSs protect against epithelial barrier dysfunction induced by cigarette smoke extract^13^. Therefore, we investigated the effects on ISE-induced TEER reduction in Calu-3 cells after pretreatment with vehicle, 10 nM GCSs (FP or BUD), and/or 10 nM LABAs (SAL or FOR) for 2 h before exposure to 50% ISE (plus GCS/LABA) for an additional 24 h. However, we found no effects of GCSs and LABAs, alone or in combination, on ISE-induced reduction in TEER (Fig. 5a–c).

### The antioxidant NAC protects against ISE-induced impairment of bronchial epithelial barrier integrity

**Figure 6:**
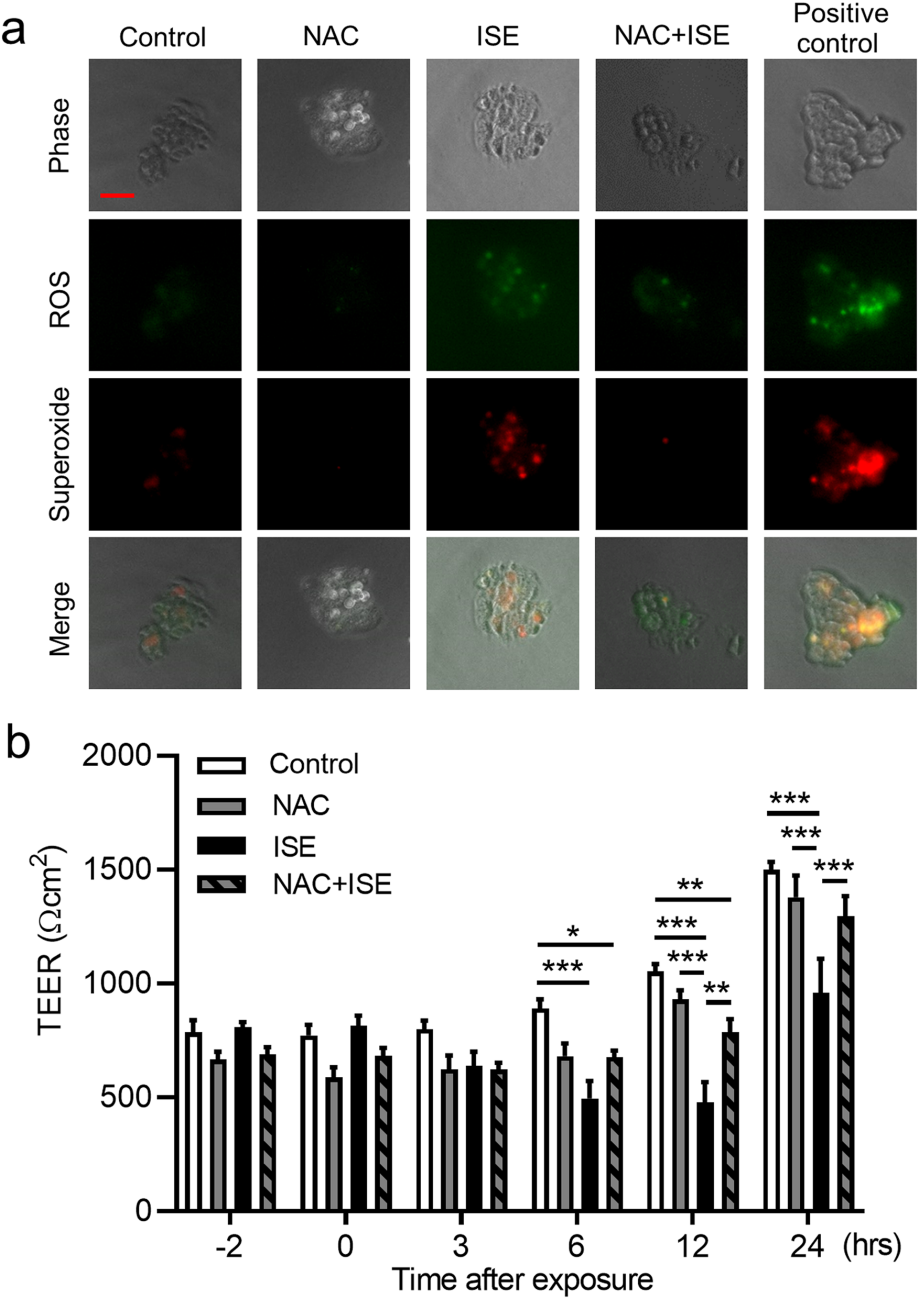
Effects of NAC on ISE-induced ROS generation and TEER reduction in Calu-3 cells. Cells were pretreated with 1 mM NAC or vehicle for 2 h and then incubated with vehicle or 50% ISE. (**a**) Detection of total ROS (green) or superoxide (red) in Calu-3 cells at 1 h after exposure to ISE. Scale bar, 50 μm. Images are representative of two independent experiments (n = 3 in each group per experiment). (**b**) Analysis of TEER at the indicated times before and after exposure to ISE. Data represent the mean ± SEM (n = 8–15 per group) and were pooled from four independent experiments. *p < 0.05, **p < 0.01, ***p < 0.001 by one- or two-way ANOVA as appropriate. *ISE* incense smoke extract, *NAC* *N*-acetyl-l-cysteine, *ROS* reactive oxygen species, *TEER* transepithelial electrical resistance.

Immunofluorescence staining of Calu-3 cells confirmed that generation of total ROS, including superoxide, could be detected within 1 h of exposure to 50% ISE, and pretreatment of cells with 1 mM NAC for 2 h before ISE exposure diminished ROS generation (Fig. 6a). Similarly, NAC pretreatment strongly suppressed the decrease in TEER observed at 12 and 24 h after Calu-3 cells exposure to 50% ISE (Fig. 6b), suggesting that ROS production was directly involved in ISE-induced disruption of epithelial barrier function. Treatment of ISE-exposed or unexposed cells with NAC alone did not affect cell viability (Supplementary Fig. S4).

### NAC treatment prevents disruption of TJ-associated proteins in mouse lungs exposed to IS

**Figure 7:**
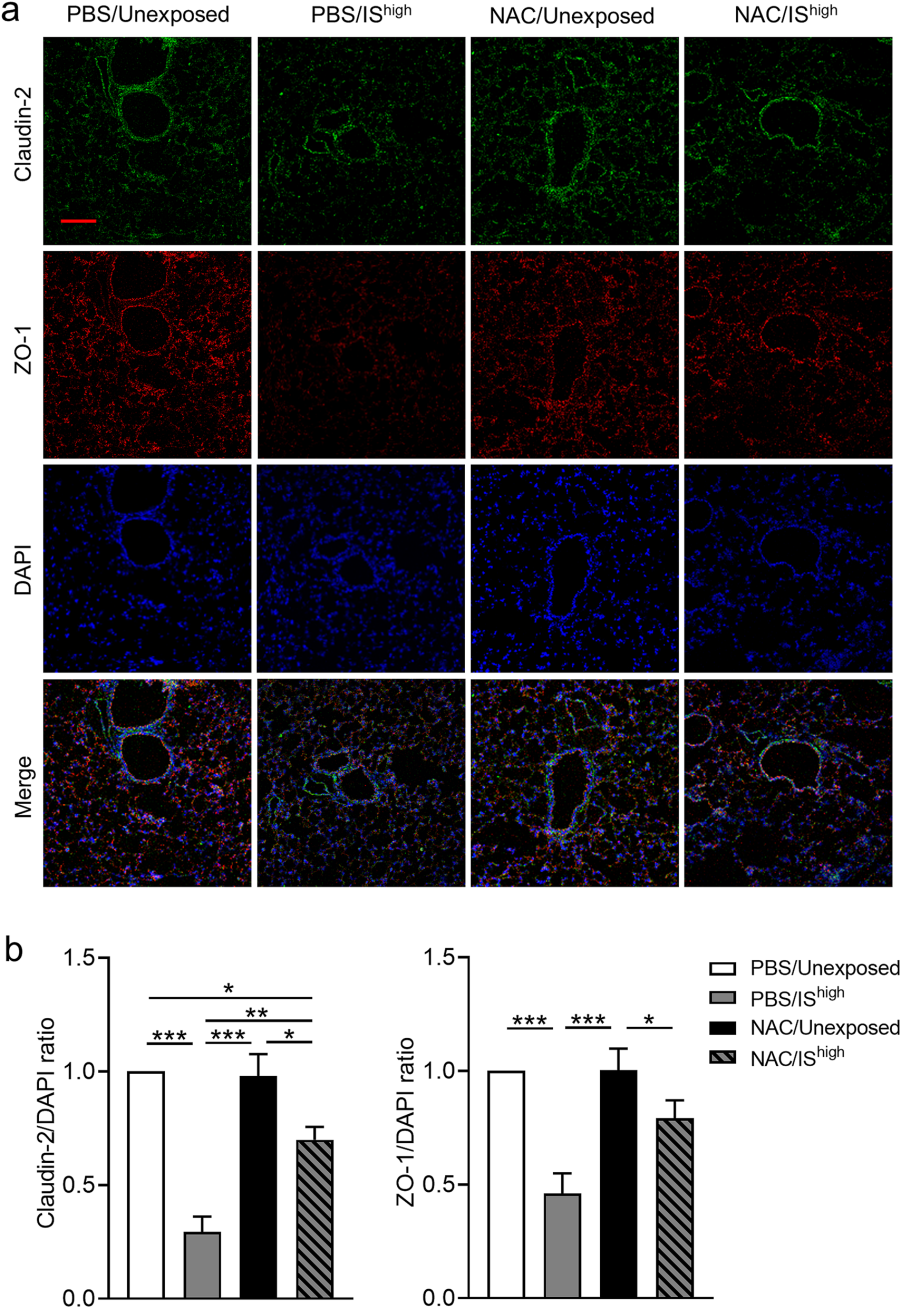
Effects of NAC on IS-induced changes in TJ-associated protein expression in mouse lungs. (**a**) Confocal immunofluorescence microscopy of ZO-1 (red) and claudin-2 (green) proteins in lung sections prepared 24 h after a 1-h exposure to fresh air (unexposed) or IS^high^. Mice were administered NAC (320 mg/kg) or PBS (vehicle) by intraperitoneal injection 6 h prior to and 6 h after the 1-h IS exposure. DAPI staining of nuclei is shown in blue. Scale bar, 100 μm. Images are representative of two independent experiments (n = 3 in each group per experiment). (**b**) Immunofluorescence intensity of Claudin-2, ZO-1 and DAPI was quantified using ImageJ and the relative quantity of Claudin-2 or ZO-1 was plotted against DAPI and normalized by unexposed control. Data represent the mean ± SEM (n = 8 per group) and were pooled from two independent experiments. *p < 0.05, **p < 0.01, ***p < 0.001 by one-way ANOVA. *DAPI* 4′,6-diamidino-2-phenylindole, *IS* incense smoke, *NAC* *N*-acetyl-l-cysteine, *PBS* phosphate-buffered saline.

To determine whether NAC can also protect lungs against IS exposure in vivo, we examined TJ-associated protein expression in lung tissues. Immunofluorescence staining of claudin-2 and ZO-1 was performed on lung sections collected at 24 h after exposure. As noted earlier, IS^high^ exposure caused discontinuous or attenuated staining of claudin-2 and ZO-1 in both bronchial epithelial and alveolar cells (Fig. 3 and Supplementary Fig. S2). Although 24 h of NAC treatment alone did not affect the pattern or intensity of protein expression in unexposed mice, NAC treatment effectively prevented the IS^high^-induced disruption of claudin-2 and ZO-1 immunostaining patterns (Fig. 7a). IS exposure-induced reduction in immunofluorescence intensity of claudin-2 and ZO-1 was significantly attenuated by NAC treatment (Fig. 7b).

### NAC treatment prevents IS-induced AHR and inflammation in mouse lungs

**Figure 8:**
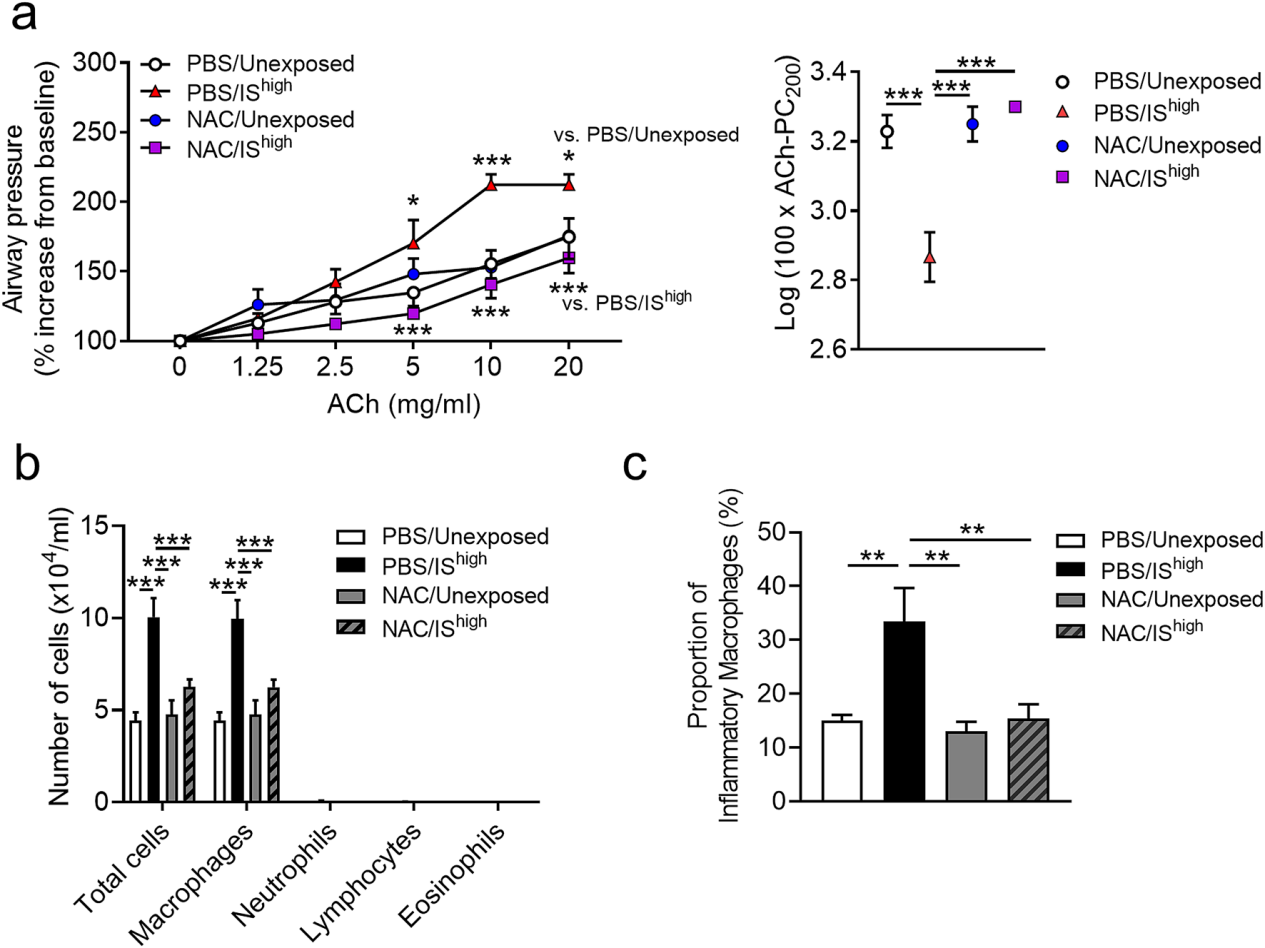
Effects of NAC on IS-induced AHR and inflammation in mouse lungs. Mice were administered NAC (320 mg/kg) or vehicle intraperitoneally 6 h prior to and 6 h after a 1-h exposure to fresh air (unexposed) or IS^high^ and evaluated 24 h later. (**a**) Acetylcholine-induced AHR. (**b**) Total and differential cell counts in BALF. (**c**) Flow cytometry analysis of macrophage subsets in the lungs. Data represent the mean ± SEM (n = 5–7 per group) and were pooled from two independent experiments. *p < 0.05, **p < 0.01, ***p < 0.001 by one- or two-way ANOVA as appropriate. *ACh* acetylcholine, *AHR* airway hyperresponsiveness, *IS* incense smoke, *NAC* *N*-acetyl-l-cysteine, *PBS* phosphate-buffered saline, *PC*_*200*_ provocative concentration causing 200% response.

Finally, we examined the effects of NAC on IS-induced AHR and accumulation of inflammatory macrophages in BALF of mice at 24 h after IS exposure. NAC treatment of IS^high^ mice reversed the effect of IS on acetylcholine-induced AHR to the same levels detected in unexposed mice (Fig. 8a). Similarly, NAC treatment completely abrogated both the increase in macrophage abundance and the proportion of inflammatory macrophages in BALF after exposure to IS^high^ (Fig. 8b, c).

## Discussion

In the present study, we showed that a single exposure of mice to IS aggravated AHR, provoked an influx of inflammatory macrophages into the lungs, and disrupted the expression and location of TJ-associated proteins in bronchial epithelium. In vitro experiments confirmed that exposure of bronchial epithelial cells to ISE induced ROS production and dose-dependently reduced TEER through a mechanism resistant to clinically relevant concentrations of GCSs and/or LABAs. Finally, NAC treatment ameliorated IS-induced effects on AHR, macrophage recruitment and claudin-2 and ZO-1 expression in the murine airway and reversed the effect of ISE on TEER in vitro.

Our results suggest that even a brief exposure to IS can disrupt epithelial barrier function and potentially exacerbate asthma in a manner resistant to treatment with inhaled GCSs. Other studies have demonstrated that long-term exposure to IS induces histological changes in the lungs in various animal models. Exposure of male rats to IS for 14 weeks at a rate of 4 g/day induced ultrastructural changes in alveolar pneumocytes, neutrophil infiltration in pulmonary alveoli, degenerative and necrotic changes, and deposition of collagen fibrils in alveolar wall^20^. However, Gaschler et al*.* showed that mice exposed to cigarette smoke (5 days a week for 8 weeks) have an increase in the number of cells in BALF, with macrophages representing greater than 95% of cells, similar to our observations^21^. Rabah et al*.* reported that repetitive exposure to IS caused histological changes in murine lungs in a dose- and duration-dependent manner^22^. These data suggest that induction of neutrophilic inflammation in lungs depends on the duration of exposure, and this may be why no increase in inflammatory cells in BALF except for macrophages was observed in our study.

The results in the present study indicate that oxidative stress may be responsible for IS-induced respiratory complications by inducing AHR, disassembly of TJ proteins, and epithelial barrier integrity. Other investigators also showed that exposure to IS induced oxidative stress and inflammatory response in vivo and in vitro^23–27^. Previous reports suggest that increased ROS may be associated with aggravated AHR and impaired epithelial barrier function. Using animal models, ROS has been shown to contribute directly to AHR via damage to oxidant-sensitive β-adrenergic receptors and increases in vagal tone, and this effect was enhanced when the epithelium was injured^28–30^. Studies of cigarette smoke exposure in bronchial epithelium suggest that ROS production after exposure may induce disassembly of TJ proteins and impair epithelial barrier function through epidermal growth factor receptor (EGFR)-extracellular signal-regulated kinase 1/2 signaling pathway^31,32^. Furthermore, Heijink et al. reported that the protective effect of GCSs against cigarette smoke induced-epithelial barrier dysfunction was mediated by affecting EGFR-downstream target glycogen synthase kinase-3β^33^. We also showed that cigarette smoke-induced reduction in TEER (approximately 35% compared with untreated control) was significantly attenuated in Calu-3 cells treated with 10 nM BUD (15.6% TEER reduction compared with untreated control) or 10 nM FP (9.7% TEER reduction compared with untreated control)^13^. However, in the present study, IS-induced reduction in TEER (approximately 35% compared with untreated control) was affected by neither BUD nor FP. Together with those observations, our present results suggest that IS-induced ROS may impair epithelial barrier integrity through a different signal transduction pathway activated by cigarette smoke exposure and IS-induced barrier dysfunction might be resistant to treatment with inhaled GCSs.

In this study, we showed that IS exposure disrupted claudin-2 and ZO-1 and simultaneously downregulated the gene expression of multiple TJ-associated proteins in the lung. Recent findings suggest a broad defect in adhesion mechanisms in asthma. Bronchial epithelium from patients with asthma has been shown to exhibit an irregular ZO-1 and occludin staining pattern and reduced barrier function that is further compromised by exposure to the Th2 cytokines IL-4 and IL-13^34,35^. Recent studies have identified an association between claudins and the development of asthma, which are expressed in a tissue- and cell type-selective manner and are essential for TJ formation^9^. Claudin-18 was found to be expressed at lower levels in the bronchial epithelium of asthma patients in a manner that correlated inversely with Th2 inflammation markers^12^. Plasma levels of claudin-4 and claudin-5 were reported to be elevated in asthma patients, particularly during exacerbations, and the levels were found to correlate negatively with lung function and positively with total IgE level and the percentage of blood eosinophils^36,37^. Loss of claudin-18 also induced epithelial barrier dysfunction in murine lungs, increased sensitisation to aspergillus and increased acetylcholine-induced AHR^12^. Although claudin-2 is expressed in healthy human bronchiolar and alveolar cells, its precise function in lung physiology is unknown^38^. A limitation of our study is that the consequences of claudin-2 and ZO-1 disruption by IS exposure remain unclear. Further studies are needed to clarify the impact of these changes on the maintenance of airway responsiveness and epithelial barrier function. Another limitation is that only female mice were utilized in this study since we previously reported AHR in an ovalbumin (OVA) model of asthma using female mice^39,40^. Matsubara et al*.* showed sex differences in induction of AHR in OVA-exposed mice^41^. It reminds unclear whether exposure to IS contributes to the development of AHR and disruption of TJ-associated proteins in lungs regardless of sex.

In conclusion, our results suggest that inhalation of IS might be harmful to respiratory health, as evidenced by AHR, increased recruitment of inflammatory macrophages and disruption of TJ-associated proteins in the lung, and damage to epithelial barrier function. However, treatment with NAC reversed many of the detrimental effects of IS exposure, suggesting that antioxidants may be beneficial for the treatment of IS-related airway dysfunction.

## Supplementary Information

Supplementary Information.

## Data Availability

The data that support the findings of this study are available from the corresponding author, KK, upon reasonable request.
