# Supplementary material for: Incense smoke-induced oxidative stress disrupts tight junctions and bronchial epithelial barrier integrity and induces airway hyperresponsiveness in mouse lungs
Source: Sci Rep. 2021 Mar 31;11:7222. doi: 10.1038/s41598-021-86745-7 (PMC8012366; doi:10.1038/s41598-021-86745-7)
Supplement: Supplementary file 1 — Supplementary Information. [file 41598_2021_86745_MOESM1_ESM.pdf]

TITLE

Incense smoke-induced oxidative stress disrupts tight junctions and bronchial epithelial barrier integrity and induces airway hyperresponsiveness in mouse lungs

Norio Yamamoto[1], Keiko Kan-o[1,2]*, Miyoko Tatsuta[1,3], Yumiko Ishii[1], Tomohiro Ogawa[1], Seiji Shinozaki[1], Satoru Fukuyama[1], Yoichi Nakanishi[1] and Koichiro Matsumoto[1]

[1]Research Institute for Diseases of the Chest, Graduate School of Medical Sciences, Kyushu University, Fukuoka, Japan

[2]Department of Endoscopic Diagnostics and Therapeutics, Kyushu University Hospital, Fukuoka, Japan

[3]Department of Respiratory Medicine, National Hospital Organization Omuta National Hospital, Fukuoka, Japan

SUPPLEMENTARY METHODS

Flow cytometric analysis

For flow cytometry analysis, removed lungs without bronchoalveolar lavage were minced and single-cell suspensions were prepared. Cells were suspended in 100 μL of PBS containing 0.5% bovine serum albumin (BSA) and preincubated with anti-mouse CD16/CD32 antibody (BD Biosciences, San Jose, CA) for 15 min to prevent nonspecific binding via the Fcγ receptor. The cells were washed and suspended in 100 μL of PBS containing 0.5% BSA and the following antibodies for 30 min: peridinin-chlorophyll-protein (PerCP)/Cy5.5 anti-mouse CD45 (BioLegend, San Diego, CA), fluorescein isothiocyanate (FITC) anti-mouse CD11b (eBioscience, San Diego, CA), FITC anti-mouse CD11c (eBioscience), APC anti-mouse Ly-6C (BD Biosciences) and PE anti-mouse Ly-6G (BioLegend). Isotype controls were included as appropriate to facilitate gating of each population. Living cells were analyzed by addition of propidium iodide (PI) to samples in the first experiment and via the forward scatter (FSC) threshold in subsequent experiments. Cells were thoroughly washed and analyzed using a BD FACSVerse flow cytometer with FACSuite software (Becton Dickinson, Franklin Lakes, NJ). One hundred thousand events were acquired in list mode. Macrophages or inflammatory macrophages in lung cells were identified using the flow cytometry gating strategy outline as described previously[S1]. In short, macrophages or inflammatory macrophages were identified as

CD45^{high} CD11b/CD11c^{high} side scatter (SSC)-A^{low}Ly-6G^{low} or CD45^{high} CD11b/CD11c^{high} SSC-A^{low} Ly-6G^{low} Ly-6C^{high}, respectively.

RNA extraction, cDNA synthesis and quantitative reverse-transcriptase (RT)-PCR

Total RNA was isolated from mouse lungs using TRI Reagent (Molecular Research Centre, Inc, Cincinnati, OH). Reverse transcription was performed using Multiscribe Reverse Transcriptase (Invitrogen, Carlsbad, CA). Real-time quantitative RT-PCR analyses were performed once per sample using SYBR Premix Ex Taq II (Takara, Shiga, Japan) and a Thermal Cycler Dice Real Time System II (Takara). The expression levels were calculated from the threshold cycle according to the delta-delta Ct method. Target gene expression levels were normalized to expression of glyceraldehyde-3-phosphate dehydrogenase (GAPDH).

Air-liquid interface (ALI) cell culture

Calu-3 cells were seeded onto human collagen type IV-coated (Sigma-Aldrich, St. Louis, MO) transwell inserts (0.33-cm^2 polyester, 0.4-μm pore size; Corning Costar, Tewksbury, MA) at a density of 1×10^6 cells/cm^2 with 200 μl apical volume and 500 μl basal volume. After 24 h, the apical medium was removed and the cells were maintained with 500 μl of culture medium supplemented with 10% foetal bovine serum and 1% penicillin-streptomycin in the

basal chamber as described previously[S2]. The basal medium was changed every other day and the monolayers were allowed to differentiate under the ALI condition for 9 days.

Permeability assay

Fluorescein isothiocyanate (FITC)-dextran (4kDa, Sigma-Aldrich) was diluted in culture medium to a concentration of 1 mg/ml. A hundred μl of medium containing FITC-dextran and 500 μl of medium were added to the apical and basal chamber, respectively. Then cells were incubated for 2 h and then culture medium in basal chamber were collected to measure fluorescence using fluorometer (Flexstation3, Molecular Devices, Tokyo, Japan). The excitation and emission wavelengths were 488 and 525 nm, respectively. A fluorescent standard curve was generated using known concentration of FITC-dextran in culture medium.

REFERENCES

S1 Fujita, A. *et al.* Inhibition of PI3Kdelta Enhances Poly I:C-Induced Antiviral Responses and Inhibits Replication of Human Metapneumovirus in Murine Lungs and Human Bronchial Epithelial Cells. *Front Immunol* **11**, 432, doi:10.3389/fimmu.2020.00432 (2020).

S2 Kreft, M. E. *et al.* The characterization of the human cell line Calu-3 under different

culture conditions and its use as an optimized in vitro model to investigate bronchial epithelial function. *Eur J Pharm Sci* **69**, 1-9, doi:10.1016/j.ejps.2014.12.017 (2015).

Table S1: Primer sequences used for qRT-PCR in this study.

Claudin-1	Sense: 5′ CCCCCATCAATGCCAG 3′
	Antisense: 5′ GGCTTGGGGATAAGGCC 3′
Claudin-2	Sense: 5′ AAAACGGAGCCGTCCT 3′
	Antisense: 5′ TTGGACCCCGTTCGCC 3′
Claudin-3	Sense: 5′ CAGTGTACCAACTGCG 3′
	Antisense: 5′ ACCGGTACTAAGGTGA 3′
Claudin-7	Sense: 5′ CTTTGCTTTCACTGCCTGGACA 3′
	Antisense: 5′ ACGCCCATGAACGTTAAGTACGAG 3′
Claudin-10b	Sense: 5′ TGGGTGCTAGTGTCTTCCACACTG 3′
	Antisense: 5′ GAATCGGTAACGCAGATCTTCCAC 3′
Claudin-12	Sense: 5′ CAGACCAGTGTGTACTCAGACTTTCTACCC 3′
	Antisense: 5′ GAAGCAACATACTGACTGTCTCCTGACG 3′
Claudin-15	Sense: 5′ GCAGGGACCCTCCACATACTTG 3′
	Antisense: 5′ AGTTCATACTTGGTTCCAGCATACAGTG 3′
Claudin-18	Sense: 5′ ATGGGTGGCATGGTGCAGAC 3′
	Antisense: 5′ TGTCATCTGGTGTCAGGCCA 3′
Occludin	Sense: 5′ GGACCCTGACCGCTATGAAACAGACTAG 3′
	Antisense: 5′ ATAGGTGGATATTCCCTGACCCAGTC 3′
E-cadherin	Sense: 5′ ATTTTTCCCTCGACACCCGAT 3′
	Antisense: 5′ TCCCAGGCGTAGACCAAGA 3′
ZO-1	Sense: 5′ AGCTCATAGTTCAACACAGCCTCCAG 3′
	Antisense: 5′ TTCTTCCACAGCTGAAGGACTCACAG 3′
Gapdh	Sense: 5′ CATGGCCTTCCGTGTCCTA 3′
	Antisense: 5′ GCGGCACGTCAGATCCA 3′

Figure S1: Effects of IS exposure on the proportion of neutrophils in mouse lungs.

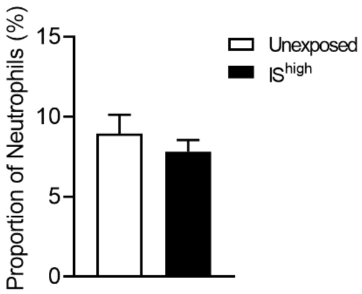

Mice were unexposed or exposed for 1 h to high doses of IS and the proportion of neutrophils in CD45-positive cells in lung-tissue-derived cells was analyzed using flow cytometry 24 h later. Data are presented as the mean ± SEM (n=6 per group) and were pooled from two independent experiments. Differences in data were analyzed by the Mann–Whitney U-test. IS, incense smoke.

Figure S2: IS exposure-induced changes in TJ and AJ-associated protein expression in mouse lungs.

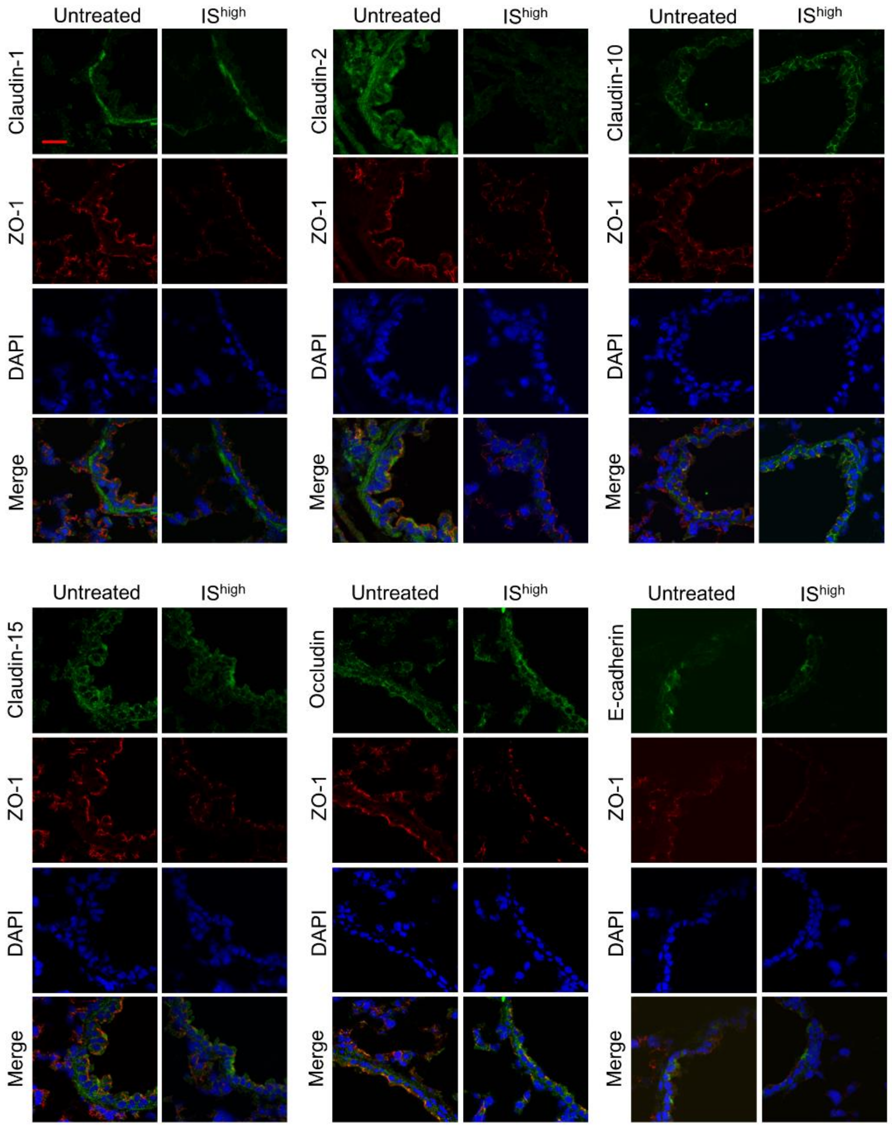

Confocal immunofluorescence microscopy of ZO-1 (red) and additional AJ and TJ-associated proteins (green) in lung sections was performed at 24 h after a 1-h exposure to fresh air (unexposed) or IS^{high}. DAPI staining of nuclei is shown in blue. Scale bar, 20 μm. Results are representative of two independent experiments (n=3 in each group per experiment). DAPI, 4′,6-diamidino-2-phenylindole; IS, incense smoke.

Figure S3: Quantification of immunofluorescence intensity of TJ and AJ-associated protein expression in mouse lungs.

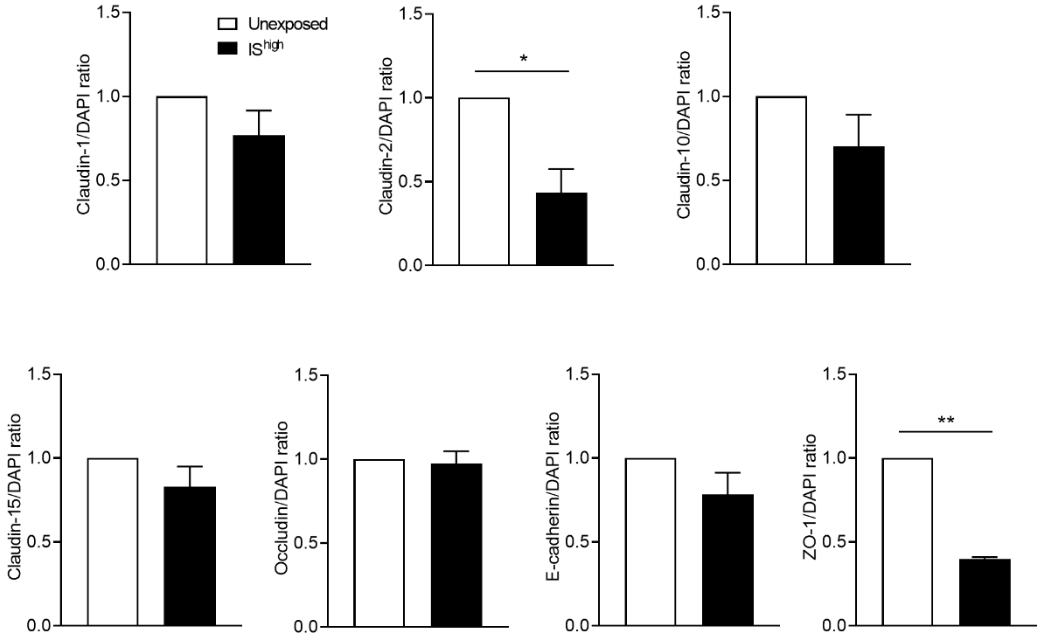

Confocal immunofluorescence microscopy of AJ and TJ-associated proteins in lung sections was performed at 24 h after a 1-h exposure to fresh air (unexposed) or IS^{high}. Immunofluorescence intensity of TJ and AJ-associated proteins and DAPI was quantified using ImageJ and the relative quantity of TJ and AJ-associated proteins was plotted against DAPI and normalized by unexposed control. Data represent the mean ± SEM (n=6 per group) and were pooled from two independent experiments. *p<0.01, **p<0.001 by the Mann–Whitney U-test. IS, incense smoke; DAPI, 4′,6-diamidino-2-phenylindole; IS, incense smoke.

Figure S4: Effects of ISE and/or NAC on cell viability.

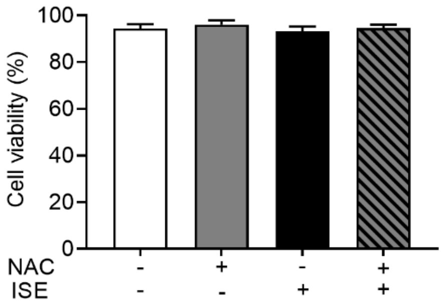

Cells were pretreated with 1 mM NAC or vehicle for 2 h and then incubated with vehicle or 50% ISE. Trypan blue cell viability assay performed 24 h after exposure to ISE. Data represent the mean ± SEM (n=4–7 per group) and were pooled from two independent experiments. Differences in data were analyzed by one-way ANOVA. NAC, N-acetyl-L-cysteine, ISE, incense smoke extract.
